# Machine learning to predict adverse perinatal outcomes: a systematic review and meta-analysis

**DOI:** 10.1016/j.eclinm.2026.104138

**Published:** 2026-08-07

**Authors:** Jingqi Zhang, Nadia Daniel, Agnese Tiranti, Xuan Li, Abigail Robinson, Shishir Rao, Marian Knight, Joris Hemelaar

**Affiliations:** a Chinese Academy of Medical Sciences Oxford Institute, University of Oxford, Oxford, UK; b National Perinatal Epidemiology Unit, University of Oxford, Oxford, UK; c Shanghai Ji Ai Genetics and IVF Institute, Obstetrics and Gynaecology Hospital, Fudan University, Shanghai, China; d Medical Sciences Division, University of Oxford, Oxford, UK; e Cancer Epidemiology Unit, Nuffield Department of Population Health, University of Oxford, Oxford, UK; f Clinical Trial Service Unit and Epidemiological Studies Unit, Nuffield Department of Population Health, University of Oxford, Oxford, UK; g Nuffield Department of Women’s & Reproductive Health, University of Oxford, Oxford, UK; h Infectious Disease Epidemiology Unit, Nuffield Department of Population Health, University of Oxford, Oxford, UK

**Keywords:** Machine learning, Prediction, Preterm birth, Small for gestational age, Stillbirth

## Abstract

**Background:**

Machine learning (ML) provides a promising approach to predict adverse perinatal outcomes, supporting early intervention to reduce neonatal morbidity and mortality. There has been a surge of publications using ML algorithms applied to routine clinical data to predict adverse perinatal outcomes. We aimed to assess the current evidence regarding ML model performance, their comparison with logistic regression, and risk of bias.

**Methods:**

We searched PubMed, EMBASE, CINAHL, Global Health, Web of Science and IEEE Xplore for studies published from 1^st^ January 2015 to 17^th^ January 2026. We included studies reporting ML models to predict preterm birth (PTB), small for gestational age (SGA) or stillbirth based on routinely collected clinical data. ML model performance was assessed mainly according to AUC based on internal validation. Study quality was assessed using PROBAST + AI. The systematic review is registered with PROSPERO, CRD42024627164.

**Findings:**

We retrieved 38,322 studies, and 90 studies were included. Overall ML model performance was moderate, with a median average AUC based on internal validation of 0.73 (range 0.50–0.93) for PTB, 0.68 (0.62–0.85) for SGA, and 0.75 (0.57–0.98) for stillbirth. The best-performing ML models showed no significant difference in AUC compared with the best logistic regression models for predicting PTB (p = 0.07) or SGA (p = 0.31), but a higher AUC for stillbirth prediction (p = 0.03). No specific or universally strong predictors were identified across models. Almost all studies were at high risk of bias, and potential publication bias was observed.

**Interpretation:**

ML prediction models showed moderate performance, were at high risk of bias and did not always outperform traditional statistical methods. To improve perinatal outcome prediction, methodological improvements and better predictors are needed.

**Funding:**

Chinese Academy of Medical Sciences Innovation Fund for Medical Science, National Institute for Health and Care Research Senior Investigator Award.

**Research in context**

**Evidence before this study**

Perinatal disorders remain a leading cause of disability-adjusted life years globally. However, most adverse perinatal outcomes occur in women without obvious risk factors who are stratified as low risk by current clinical assessment, emphasising the need for better prediction tools to support early intervention. Evidence on the performance of machine learning (ML) algorithms to predict adverse perinatal outcomes remains limited and conflicting. We searched PubMed, EMBASE, CINAHL, Global Health, Web of Science and IEEE Xplore for studies published from 1^st^ January 2015 to 17^th^ January 2026, with no restriction on languages or article types. We used both free text and controlled vocabulary search terms for (“preterm birth” OR “small for gestational age” OR “stillbirth”) AND (“machine learning” OR “prediction model”). We found no systematic review that provided quantitative comparisons between ML and traditional statistical methods using structured health data for adverse perinatal outcome prediction.

**Added value of this study**

To our knowledge, this is the largest systematic review on ML for predicting adverse perinatal outcomes based on routinely obtained clinical data. Three quarters of included studies were published since 2022, when the literature search of most recent previous systematic review was undertaken. This is also the first systematic review to quantitatively compare the performance of ML and traditional statistical methods based on structured health data in this field. We found that the overall performance of ML models was moderate. The best-performing ML models showed no significant difference in area under the receiver operating characteristic curve (AUC) compared with the best logistic regression models for predicting PTB or SGA, but a higher AUC for stillbirth prediction. This was the first review to apply the updated PROBAST + AI tool and found that almost all studies were at high risk of bias. No specific or universally strong predictors were identified across models.

**Implications of all the available evidence**

Our results indicate moderate performance, high risk of bias, and lack of evidence that ML consistently outperforms traditional statistical methods in prediction models for adverse perinatal outcomes based on routine clinical data. Future efforts should shift from repetitive model development on different datasets to strengthening methodological rigour, exploring innovative methods and novel predictors, and ensuring that models are appropriately validated and prepared for clinical implementation.

## Introduction

Perinatal disorders were the leading cause of disability-adjusted life years during 2010–2020, and remain the third leading cause in the post-pandemic period.^1^ Sustainable Development Goal (SDG) target 3.2 aims to reduce neonatal mortality to 12 or fewer deaths per 1000 live births and under-five mortality to 25 or fewer deaths per 1000 live births by 2030. Despite global progress, a large number of countries, mainly concentrated in sub-Saharan Africa and in Central and Southern Asia, are not on track to reach these targets by 2030.^2^ Adverse perinatal outcomes that are major contributors to neonatal morbidity and mortality include preterm birth (PTB) and small for gestational age (SGA).^3^ In addition, stillbirth is a major contributor to perinatal mortality.^3^ Enhanced efforts are urgently required for high-quality antenatal and childbirth care programmes to prevent these adverse perinatal outcomes.

Most adverse pregnancy outcomes occur among women who do not have obvious risk factors and who are classified as low risk according to current clinical assessment. It has been estimated that approximately two-thirds of PTBs occur in the absence of known risk factors, emphasising the pressing need for better tools to improve prediction of at-risk populations.^4^^,^^5^ Reliable predictive capabilities will enable earlier interventions and preventive strategies, thereby facilitating the attainment of the SDG goals.

Machine learning (ML) provides a promising approach to model complex nonlinear relationships between maternal characteristics, clinical measurements and pregnancy outcomes captured in high-dimensional data from electronic health records and registry databases.^6^, ^7^, ^8^ Although extensive analyses of individual aetiological factors have been undertaken, the components of potential risk factors, interactions among risk factors and the pathophysiology of these adverse perinatal outcomes remain largely unclear.^9^ In this context, ML classifiers, such as support vector machine, random forests and decision trees that learn from structured data to classify outcomes, have been applied to disease prediction across various fields, demonstrating impressive performance and being considered complementary or even superior to traditional statistical methods.^10^ However, the implementation of ML prediction models in the field of perinatal health care is currently limited by inadequate reporting and uncertain performance in existing studies, which may lead to inappropriate antepartum and intrapartum management.^11^^,^^12^

Early systematic reviews often included a variety of outcomes or mixed data types, or lacked systematic assessment of risk of bias.^13^, ^14^, ^15^ Systematic reviews that focused only on specific perinatal outcomes prediction by ML methods using routinely collected clinical data were typically small-scale and, more importantly, did not provide detailed quantitative analyses to support conclusions on the effectiveness of ML compared to traditional statistic methods.^15^ Moreover, the most recent systematic review on this topic conducted its literature search in 2022; however, in the past three years, there has been an accelerated rise in publications of ML in obstetrics, revealing a significant gap in the current analyses for these ML models.^16^ Therefore, we aimed to update the evidence for adverse perinatal outcome prediction by ML methods, and compare their performance with logistic regression. We focused on supervised ML classifiers based on structured data that are routinely available in current clinical practice, and limited our analysis to the most important adverse perinatal outcomes, PTB, SGA and stillbirth, aiming to reduce heterogeneity across studies and provide interpretable results.

## Methods

### Search strategy

We performed this systematic review according to the PRISMA 2020 guidelines.^17^ The study protocol was registered on PROSPERO (CRD42024627164). We performed a comprehensive search in six electronic databases including PubMed, EMBASE, CINAHL, Global Health, Web of Science and IEEE Xplore. We used both free text and controlled vocabulary search terms for (“preterm birth” OR “small for gestational age” OR “stillbirth”) AND (“machine learning” OR “prediction model”) (Appendix pp 2–6). Publications from 1^st^ January 2015 to 17^th^ January 2026 were retrieved with no restriction on languages or article types. We supplemented the electronic database searches with a manual search for relevant primary papers in the reference lists of included articles, relevant reviews and systematic reviews. The search results were imported into Covidence, a web-based systematic review collaboration software platform, to streamline deduplication, abstract screening, and full text review.^18^

### Eligibility criteria

We included studies if they met all of the following criteria: (1) the study population consisted of a general population of pregnant women, irrespective of whether the outcome was a live birth or stillbirth; (2) the study described at least one ML model designed to predict the occurrence of PTB (birth before 37 weeks of gestation), SGA (birthweight below the 10^th^ centile according to any chart) or stillbirth (delivery of an infant without any signs of life after at least 20 weeks of gestation); (3) the model was developed or validated based on routinely obtained clinical data; (4) model performance was quantitatively evaluated using at least one relevant metric, such as AUC, accuracy, sensitivity, specificity or any other quantitative outcome metrics.

Studies were excluded if: (1) the study population exclusively consisted of pregnant women with specific medical conditions or high-risk subgroups (e.g., those diagnosed with maternal heart disease, or premature rupture of membranes); (2) the prediction target was limited to newborns with specific complications or abnormalities (e.g., neonates with neurodevelopment impairment); (3) the ML approach employed data types such as omics data, electrical signal analysis, or image recognition rather than clinical variables; (4) the publication was a review, meta-analysis, conference abstract, letter, or commentary; or (5) the study was based on animal experiments. Screening of abstracts and full text review for eligibility were conducted by one primary reviewer (J.Z.) and four secondary reviewers (N.D., A.T., A.R., and X.L.). Each record was independently assessed by two of the reviewers.

### Data extraction

We extracted data into pre-designed and standardised data extraction forms, including study characteristics, data source, model development methods, predictors and model performance. Data for each study were extracted by one primary reviewer (J.Z.) and four secondary reviewers (N.D., A.T., A.R., and X.L.). Disagreements were resolved by discussion until consensus was reached or by consulting senior investigators (M.K. and J.H.).

### Risk of bias assessment

Two reviewers independently assessed the quality of model development and the risk of bias of model evaluation using PROBAST + AI (Prediction Model Risk of Bias ASsessment Tool + Artificial Intelligence).^19^ PROBAST + AI consists of 34 signalling questions, in four domains: 1. Participants and data sources, 2. Predictors, 3. Outcome, and 4. Analysis. Based on the answers to signalling questions, the entire domains were rated as low, high, or unclear quality concern for the model development, or low, high, or unclear risk of bias for the model evaluation. The overall assessment of quality and risk of bias was rated as low, high, or unclear. A study was classified as having an overall low risk of bias only if it was at low risk of bias within each domain, high if at least one domain was judged to be at high risk of bias, and unclear if at least one domain was judged unclear and all other domains were judged as low. Any disagreement was resolved by discussion until consensus was reached or by consulting senior investigators.

### Data synthesis

We summarised the extracted data using a combination of narrative synthesis, descriptive statistics and graphs. We analysed study characteristics, data source, predictor sets and model development approaches by outcome of PTB, SGA and stillbirth.

As most studies developed multiple predictive models, and intra-study variability was typically smaller than inter-study variability, we chose the average mean area under the receiver operating characteristic curve (AUC) to represent the performance of each study. Unless specified, all analyses were based on internal validation in order to ensure the robustness of measurement, compared to the apparent performance. The best-performing model was determined through a joint assessment of reported performance metrics, including AUC, accuracy, sensitivity and specificity, with AUC considered the principal measure of discrimination, as it is a threshold-independent metric.

### Statistical analysis

To compare the performance of the best-performing ML and logistic regression models within the same study, we applied the Wilcoxon signed-rank test to studies that reported AUCs for both methods. We also generated a paired difference plot to visualise the differences between ML and logistic regression models within each study.

As exploratory analyses, we also performed random-effects meta-analyses separately for each outcome. The effect size was defined as the difference in discriminative performance between ML and logistic regression models, expressed as ΔAUC. To further compare the performance of different ML algorithms, we performed three-level random-effects meta-regression with logit-transformed AUCs. If a study reported multiple models using the same algorithm, only the model with the highest AUC was retained. Differences between algorithm families were assessed using an omnibus Wald test. Study-specific variances were estimated using the method of Hanley and McNeil.^20^ Pooled estimates were obtained using restricted maximum likelihood estimation with separate between-study and within-study variance components to account for multiple algorithm estimates reported within the same study. Heterogeneity was quantified using *I*^*2*^ and τ^2^ statistics.

We constructed funnel plots to investigate the potential for publication bias. We further conducted Egger’s regression tests to explore funnel plot asymmetry and potential small-study effects for each outcome, with p < 0.05 suggesting possible publication bias.^21^

All data synthesis and statistical analyses were performed using Python 3.11, and GraphPad Prism 10 was used to create the figures.

### Ethics statement

Ethical approval and informed consent were not required because this study is a systematic review of previously published studies.

### Role of the funding source

The sponsor of the study had no role in study design, data collection, data analysis, data interpretation, or writing of the report. The corresponding author had full access to all the data in the study and had final responsibility for the decision to submit for publication.

## Results

**Fig. 1:**
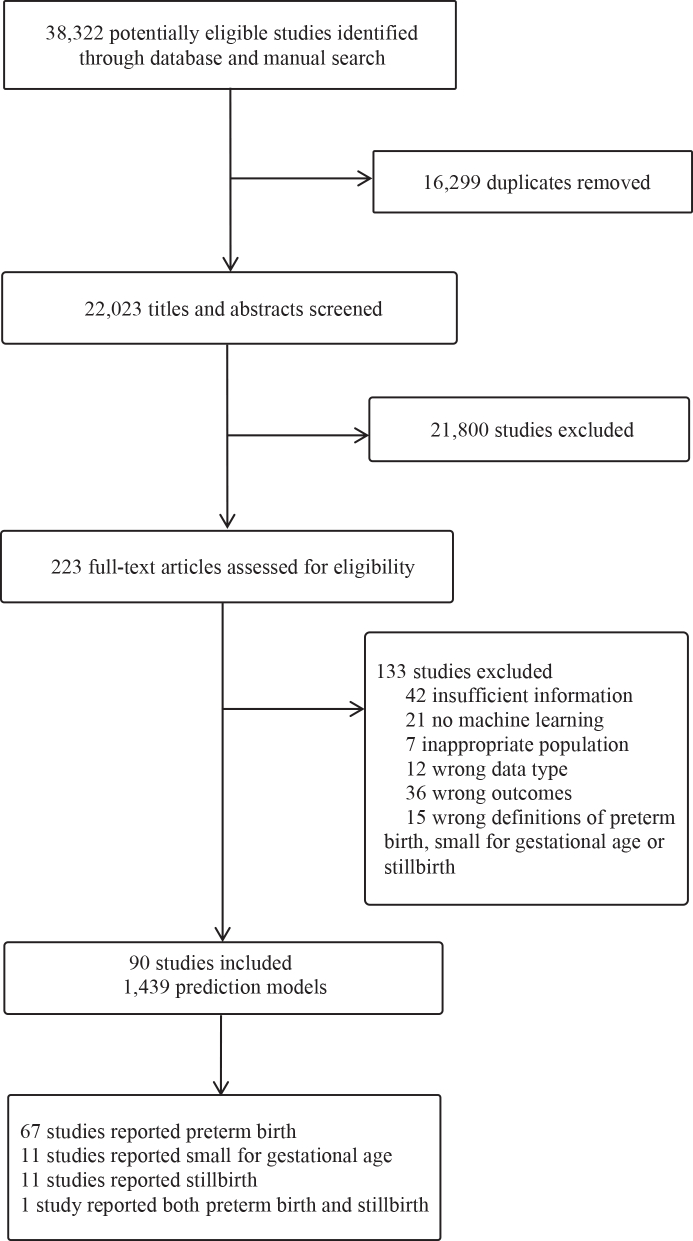
PRISMA flow diagram of studies predicting adverse perinatal outcomes using machine learning.

Our search retrieved 38,322 articles, and a total of 90 studies (Fig. 1) reporting on 1439 prediction models, including 1163 ML models, were included. Sixty-seven studies focused on PTB, 11 studies on SGA, 11 studies on stillbirth, and one study reported prediction models for both PTB and stillbirth.

The characteristics of the studies included in this review are displayed in Table 1 and Appendix pp 7–10. Most studies focused on model development, with only 12.2% (11/90) reporting external validation, and approximately half (7/11) of these employed temporal validation (Table 1). In most cases (90.0%, 81/90), data were retrospectively collected, mostly after 2010 (Table 1). Fifty-three percent (48/90) of the studies drew data from multiple sites, while the remainder relied on a single site (Appendix pp 7–9). Geographically, the majority of studies (84.4%, 76/90) were conducted in high or upper-middle-income countries. Sample size across included studies ranged from 50 to 15,976,537, with a median of 18,201 (Table 1). More than half (58.9%, 53/90) of the studies included both nulliparous and multiparous women in their model (Appendix pp 7–9). Regarding multiple pregnancies, 47.8% (43/90) studies exclusively focused on singletons, and about one-third (34/90) of studies included both singleton and multiple pregnancies in the analysis (Appendix pp 7–9).

**Table 1:** Characteristics of studies included in the systematic review.

Author year	Country	Study period	Study design	Study type	Sample size	Incidence of outcome	Prediction timing
Preterm birth							
Abdi et al. (2025)^22^	Iran	2020–2022	Retrospective	D	8853	13.9%	Around delivery
Abraham et al. (2022)^23^	US	NA	Retrospective	D&V	33,570	6.4%	Early antenatal phase, first trimester, second trimester, after birth
Alif et al. (2024)^24^	India	2018–2021	Retrospective	D	449	17.8%	After birth
Alsaad et al. (2022)^25^	US	2000–2017	Retrospective	D	222,436	8.0%	Early antenatal phase, first trimester, second trimester, third trimester
Amini et al. (2017)^26^	Iran	2015–2015	Prospective	D	4419	5.5%	NA
Begum et al. (2021)^27^	Bangladesh	NA	Retrospective	D	3500	36.2%	NA
Belaghi et al. (2021)^28^	Canada	2012–2014	Retrospective	D	112,963	6.2%	First trimester, second trimester
Belaghi et al. (2024)^29^	Canada	2012–2014	Retrospective	D	145,846	5.6%	First trimester, second trimester
Bitar et al. (2024)^30^	US	2019–2021	Prospective	D	12,071	16.4%	Second trimester
Chen et al. (2024)^31^	China	2019–2022	Retrospective	D&V	3082	44.5%	NA
Chun et al. (2025)^32^	Singapore	2017–2023	Retrospective	D	91,174	10.2%	first trimester
Deshpande et al. (2023)^33^	India	2018–2019	Retrospective	D	903	16.2%	After birth
Diaz et al. (2022)^34^	Spain	2015–2016	Retrospective	D	380	41.3%	Third trimester
Ding et al. (2024)^35^	China	2021–2022	Retrospective	D&V	66,728	7.8%	After birth
Diniz et al. (2025)^36^	Brazil	2017	Retrospective	D	5060	11.8%	Around delivery
Ebrahimvandi et al. (2022)^37^	US	2016	Retrospective	D	3,610,827	7.7%	First trimester
Esty et al. (2018)^38^	Canada and US	NA	Retrospective	D	NA	7.9%	Second trimester
Farrokhi et al. (2024)^39^	Iran	2019	Retrospective	D	145,431	8.7%	NA
Gao et al. (2025)^40^	China	2020–2024	Retrospective	D	19,818	3.3%	Second trimester
Hassan et al. (2025)^41^	Brazil		Retrospective	D	696	50.0%	Third trimester
Hershey et al. (2022)^42^	US	1992–1994	Retrospective	D	2390	8.4%	NA
Huang et al. (2024)^43^	US	2010–2013	Prospective	D	8830	8.6%	First trimester, second trimester, third trimester
Khan et al. (2023)-a^44^	United Arab Emirates	2017–2021	Prospective	D	3508	11.2%	Third trimester
Khan et al. (2023)-b^45^	United Arab Emirates	2017–2021	Prospective	D	3509	11.23%	After birth
Khatibi et al. (2019)^46^	Iran	2016–2017	Retrospective	D	1,431,597	8.1%	NA
Kim et al. (2024)^47^	US	2012–2015, 2016–2017	Retrospective	D	78,356	7.1%	Around delivery
Kim et al. (2025)-a^48^	US	2016–2021, 2014–2020	Retrospective	D	9595	19.7%	Around delivery
Kim (2025)-b^49^	US	2019	Retrospective	D	1,032,465	9.5%	First trimester
Kloska et al. (2025)^50^	Poland	NA	Retrospective	D	50	56.0%	Around delivery
Koivu et al. (2020)-a^51^	US	2013–2016	Retrospective	D&V	15,976,537	9.6%	NA
Kong et al. (2024)^52^	Greece	2022–2022	Prospective	D	715,962	6.4%	Around delivery
Kokkinidis et al. (2023)^53^	China	2014–2017	Retrospective	D	345	36.3%	NA
Lee et al. (2019)^54^	South Korea	2014–2018	Retrospective	D	596	7.2%	Second trimester
Lee et al. (2020)^55^	South Korea	1995–2018	Retrospective	D	731	16.8%	After birth
Lee et al. (2021)-a^56^	South Korea	2015–2017	Retrospective	D	NA	7.2%	Around delivery
Lee et al. (2021)-b^57^	South Korea	2015–2017	Retrospective	D	402,092	7.2%	Around delivery
Lee et al. (2022)-a^58^	South Korea	2015–2017	Retrospective	D	402,092	7.2%	Around delivery
Lee et al. (2022)-b^59^	South Korea	2015–2017	Retrospective	D	402,092	NA	Around delivery
Lee et al. (2024)^60^	South Korea	2017	Retrospective	D	489,893	NA	Around delivery
Liu et al. (2024)^61^	US	2022, 2018	Retrospective	D&V	2,567,040	NA	After birth
Marvin et al. (2022)^62^	*India*	2018–2021	Retrospective	D	450	8.0%	NA
Meem et al. (2022)^63^	Brazil	2016–2017	Retrospective	D	779	49.2%	After birth
Mirzamoradi et al. (2024)^64^	Iran	2018–2019	Retrospective	D	300	50.0%	NA
Padmavathi et al. (2024)^65^	India	NA	Retrospective	D	101,400	0.9%	After birth
Pari et al. (2017)^66^	India	NA	Retrospective	D	2600	25.5%	Around delivery
Pari et al. (2025)^67^	India	NA	Retrospective	D	2600	25.5%	Around delivery
Park et al. (2024)^68^	South Korea	NA	Prospective	D	60	50.0%	Around delivery
Pathak et al. (2025)^69^	India	NA	Retrospective	D	5400	NA	Second trimester
Pereira et al. (2015)^70^	Portugal	2012–2015	Retrospective	D	3376	7.1%	Around delivery
Pourahmad et al. (2017)^71^	Iran	2015–2015	Retrospective	D	1102	24.3%	NA
Priya et al. (2025)^72^	India	2017–2022	Retrospective	D	9521	14.5%	First trimester
Qian et al. (2025)^73^	China	2015–2024	Retrospective	D&V	36,378	6.2%	Around delivery
Raja et al. (2021)^74^	India	2018–2020	Retrospective	D	1300	23.8%	After birth
Ramalingam et al. (2019)^75^	India	NA	Retrospective	D	2600	NA	Around delivery
Safi et al. (2022)^76^	US	2001–2017	Retrospective	D	63,814	9.5%	Early antenatal phase, first trimester, second trimester
Siargkas et al. (2025)^77^	Greece	2012–2025	Retrospective	D	9805	8.7%	Second trimester
Silva et al. (2025)^78^	Brazil	2018–2021	Retrospective	D	526,368	11.0%	First trimester
Song et al. (2023)^79^	South Korea	2017	Retrospective	D	124,606	5.8%	Around delivery
Sun et al. (2022)^80^	China	2008–2018	Retrospective	D	9550	50.0%	Second trimester
Teng et al. (2025)^81^	China	2019–2022	Retrospective	D&V	2606	50.0%	Second trimester
Tran et al. (2016)^82^	Australia	2011–2015	Retrospective	D	18,836	11.0%	Second trimester
Waynforth et al. (2022)^83^	UK	2000–2001	Retrospective	D	18,201	7.5%	NA
Wong et al. (2022)^84^	Australia	1980–2015	Retrospective	D	958,729	8.6%	Around delivery
Yang et al. (2025)^85^	China	2022	Retrospective	D	1122	16.7%	Second trimester
Yu et al. (2024)^86^	China	2014–2016	Prospective	D	22,603	4.2%	First trimester, second trimester, third trimester
Zhang et al. (2022)^87^	China	2017–2020	Retrospective	D	5187	4.3%	First trimester, second trimester
Zhang et al. (2023)^88^	China	2019–2020	Retrospective	D&V	5411	11.7%	Around delivery
Zhang et al. (2024)^89^	China	2018–2023	Retrospective	D&V	24,770	12.8%	NA
Small for gestational age							
Akhtar et al. (2022)^90^	China	NA	Retrospective	D	36,173	36.7%	NA
Brown et al. (2025)^91^	Canada	1996–2021	Retrospective	D	9097	9.9%	Second trimester
Chen et al. (2024)^92^	China	2018–2023	Retrospective	D	4394	3.4%	First trimester, second trimester, third trimester, around delivery
Deng et al. (2025)^93^	Malaysia	2018–2022	Retrospective	D&V	5519	12.7%	Second trimester
Kuhle et al. (2018)^94^	Canada	2009–2014	Retrospective	D	30,705	7.9%	Early antenatal phase, second trimester
Saw et al. (2021)^95^	Singapore	NA	Retrospective	D	347	58.2%	Second trimester
Stoenescu et al. (2021)^96^	Romania	2016–2020	Prospective	D	135	NA	Second trimester, third trimester
Van et al. (2024)-a^97^	China	2015–2021	Retrospective	D	7898	5.3%	First trimester
Van et al. (2024)-b^98^	China	2015–2021	Retrospective	D	7898	5.3%	First trimester
Yu et al. (2025)^99^	China	2014–2016	Retrospective	D	22,603	4.2%	First trimester, second trimester, third trimester
Zhang et al. (2023)^100^	China	2012–2016	Retrospective	D	9972	11.3%	Around delivery
Stillbirth							
Adebanji et al. (2024)^101^	Ghana	2013–2015	Retrospective	D	6466	6.4%	After birth
Aga et al. (2025)^102^	Ethiopia	2023	Retrospective	D	549	3.1%	Around delivery
Bosschiete et al. (2023)^103^	US	2016–2021	Retrospective	V	153,432	0.4%	NA
Ferro et al. (2025)^104^	Brazil	2008–2022	Retrospective	D	231,505	3.1%	First trimester
Gunenc et al. (2025)^105^	Turkey	2015–2024	Retrospective	D	951	47.5%	Third trimester
Khatibi et al. (2021)^106^	Iran	2016–2017	Retrospective	D	1,421,125	0.4%	After birth
Kim et al. (2025)-c^107^	South Korea	2009–2020	Retrospective	D	32,953	0.9%	Early antenatal phase, first trimester, second trimester
Kintomonho et al. (2025)^108^	Benin	2020	Retrospective	D	1048	9.2%	Around delivery
Koivu et al. (2020)-b^109^	US	2014–2016	Retrospective	D	363,699	0.04%	NA
Koivu et al. (2020)-a^51^	US	2013–2016	Retrospective	D&V	15,976,537	0.6%	NA
Malacova et al. (2020)^110^	Australia	1980–2015	Retrospective	D	947,025	0.8%	First trimester, around delivery
Zhu et al. (2026)^111^	China	2019–2024	Retrospective	D	65,884	0.2%	Third trimester

D, development; NA, not available; UAE, United Arab Emirates; UK, the United Kingdom; US, the United States; V, validation, referring to external validation.

**Fig. 2:**
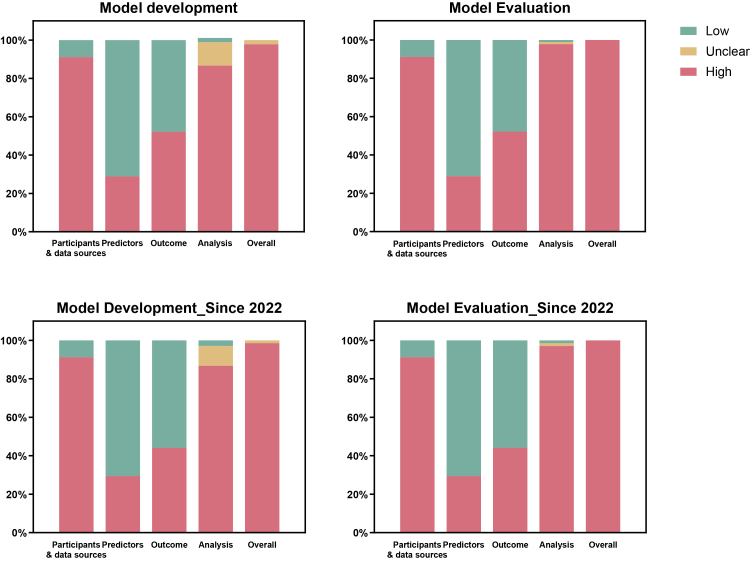
PROBAST + AI risk of bias assessment of all included studies and studies published since 2022.

Risk of bias was assessed separately for model development and model evaluation according to PROBAST + AI. In terms of model development, the overall risk of bias of almost all studies was considered high and two were considered unclear (Fig. 2, Appendix pp 10–12). For model evaluation, all studies were judged to be at high risk of bias. Regarding participants and data sources, most studies lacked sufficient details about the data source or relied on existing datasets for routine care or administrative registries. Bias in the predictor domain commonly derived from inclusion of variables unavailable at the prediction time, such as delivery method, neonatal gender or birth weight. In the outcome domain, risk was primarily due to a short time interval between predictor assessment and outcome. For model development analysis, bias was mainly related to inadequate approaches to deal with resampling, missing values and imbalanced data. Regarding model evaluation, bias was usually associated with lack of model calibration and imbalance correction. A sensitivity analysis was conducted to compare the risk of bias of studies published before and since 2022. The results showed no significant differences across periods, indicating that the methodological quality of published studies has not improved over time (Fig. 2).

Across different outcomes, the model development processes shared largely similar methodologies and analytical pipelines. Regarding the handling of missing data, many studies employed a combination of strategies (Appendix p 13). The most commonly reported approaches were imputation (41.1%, 37/90), exclusion of records with missing values in key predictors or outcomes, removal of features with high missingness, only inclusion of complete cases, and other methods (Appendix p 13). For addressing data imbalance, most studies did not specify the method used (40.0%, 36/90). Among those reported, undersampling (18.9%, 17/90) and Synthetic Minority Over-sampling Technique (18.9%. 17/90) was the most frequently applied technique, followed by oversampling, creation of balanced datasets, and class weighting methods (Appendix p 13). Nearly one third (33.3%, 30/90) of the studies did not report any data engineering procedures (Appendix p 14). Similarly, methods for hyperparameter tuning were often unspecified (30/90, Appendix p 14).

A diverse range of ML algorithms were implemented, with random forests (63.3%, 57/90) and neural networks (52.2%, 47/90) being the most frequently used (Appendix p 15). Other frequently applied techniques included boosting methods, decision tree, support vector machine, naïve Bayes, ensemble learning approaches, and a variety of other customised methods. About 63.3% (57/90) studies constructed traditional logistic regression models as baseline comparators to ML models (Appendix p 15).

**Fig. 3:**
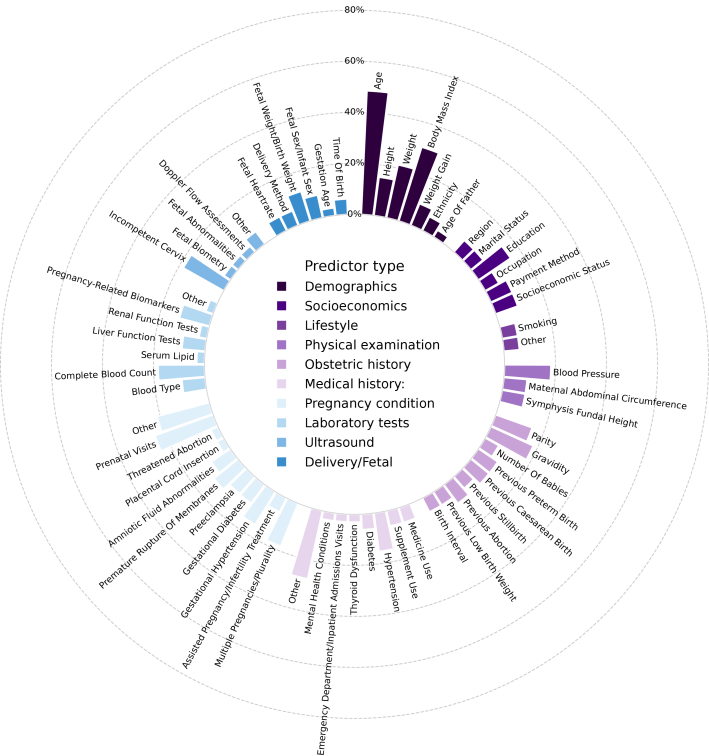
**Proportion of each predictor appearing in the top 10 most important predictors in the best-performing models across 33 studies that reported predictor rankings and model AUCs.** Abbreviations: AUC, area under the receiver operating characteristic curve.

Most studies selected predictors based on previous studies and expert knowledge, complemented by logistic regression, other statistical methods, feature importance rankings and other methods. The number of predictors included in all ML models ranged from 3 to 39,302, with a median of 22.5 predictors for the best-performing models (Appendix p 15). The most commonly included data were basic demographic information, medical history, obstetric history, and pregnancy conditions, followed by socioeconomic information and laboratory tests, while lifestyle factors, physical examinations, and ultrasound data were rarely incorporated (Appendix p 16). Among the best-performing models, the predictors most frequently included (>30% of studies) were maternal age, hypertension, parity, gestational diabetes, body mass index (BMI), diabetes, smoking, previous PTB, assisted pregnancy, pre-eclampsia, multiple pregnancy, gravidity and infection (Appendix p 17). The top ten most important features most often reported (>30% of studies) only included maternal age and BMI (Fig. 3). About 25.5% (23/90) of studies demonstrated model interpretability using SHapley Additive exPlanations (SHAP) value to provide insight into individual feature contributions. Six studies also included a decision curve analysis to evaluate the clinical utility and net benefit of the prediction models across different threshold probabilities (Appendix pp 7–9).

**Fig. 4:**
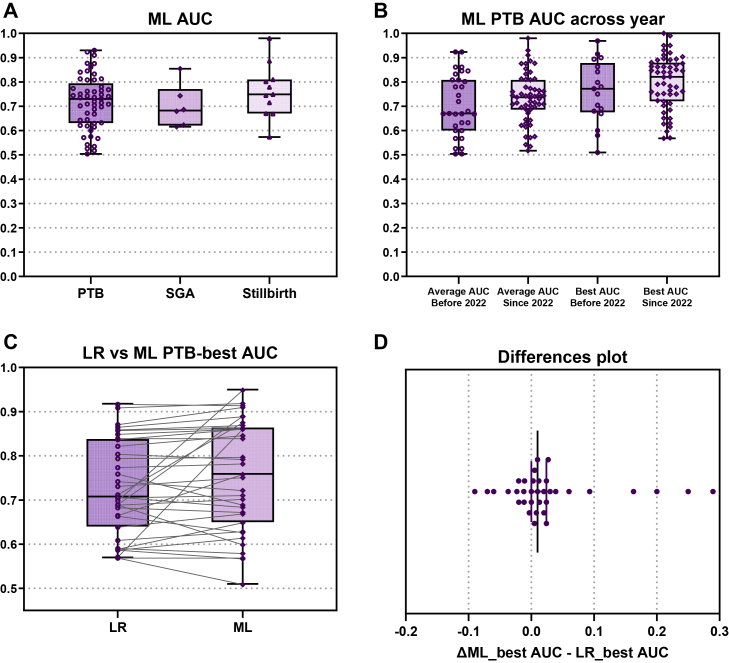
**Performance based on internal validation of predictive models for adverse perinatal outcomes.** (A) Average AUCs of ML models in each article based on internal validation. (B) Average AUCs and best AUCs of ML models for prediction of PTB in studies published before 2022 and since 2022. (C) Comparison of AUCs of best-performing models for prediction of PTB based on ML and logistic regression. (D) Paired differences in AUCs of best-performing models for prediction of PTB based on ML and logistic regression. Abbreviations: AUC, area under the receiver operating characteristic curve; ML, machine learning; LR, logistic regression; PTB, preterm birth; SGA, small for gestational age.

The median average AUC of ML prediction models based on internal validation for PTB was 0.73 (range 0.50–0.93, Fig. 4A), with a corresponding median average accuracy of 0.80 (range 0.59–0.99, Appendix p 18). Median average sensitivity was 0.66 (range 0.10–0.99, Appendix p 18). With regard to SGA, the median average AUC across models was 0.68 (range 0.62–0.85) (Fig. 4A). Median average accuracy reached 0.69 (range 0.57–0.87), and sensitivity was 0.68 (range 0.47–0.96) (Appendix p 18). In stillbirth prediction, the median average AUC was 0.75 (range 0.57–0.98) (Fig. 4A), with accuracy and sensitivity medians of 0.88 (range 0.63–0.96) and 0.62 (range 0.23–0.98), respectively (Appendix p 18). A sensitivity analysis was also conducted to compare the average and best AUCs of PTB prediction models reported in studies published before and since 2022. Average and best AUCs appeared higher since 2022 than before 2022, although these trends were not statistically significant (Fig. 4B, average AUCs: p = 0.25, best AUCs: p = 0.25).

Among studies that incorporated predictors from a later stage of pregnancy, such as in the third trimester, around delivery or after birth, the best-performing model consistently appeared in models using these late-stage variables. In 20.0% (18/90) of studies, models based on the second trimester demonstrated the best performance, while only 11.1% (10/90) of studies identified first-trimester models as the best-performing ones (Appendix p 18).

The best AUCs of ML and logistic regression models were compared across 48 articles which reported AUCs for both methods (Fig. 4C). For the prediction of PTB and SGA, no significant differences in AUC were observed between the two methods within the same studies (PTB: median ΔAUC = 0.01, p = 0.07; SGA: median ΔAUC = 0.01, p = 0.31). For stillbirth, the best-performing ML models achieved higher AUCs than the best logistic regression models (median ΔAUC = 0.02, p = 0.03). However, the robustness of this finding is limited by the small number of studies available for stillbirth. In exploratory random-effects meta-analysis of PTB studies, ML models showed a statistically significant improvement in discriminative performance compared with logistic regression (pooled ΔAUC = 0.03, 95% CI 0.01–0.06; p = 0.02). However, given the small absolute difference and high heterogeneity (*I*^*2*^ = 99.4%), the clinical improvement remains uncertain (Appendix pp 19–20). For SGA, no significant difference was observed (pooled ΔAUC = 0.01, 95% CI −0.004 to 0.03; p = 0.14), with moderate heterogeneity (*I*^*2*^ = 53.8%) (Appendix pp 19–20). For stillbirth, the pooled effect did not reach statistical significance (pooled ΔAUC = 0.04, 95% CI −0.13 to 0.22; p = 0.08), and heterogeneity was high (*I*^*2*^ = 93.3%) (Appendix pp 19–20). There was little evidence that the performance differed substantially between ML algorithms. For PTB and stillbirth, there was no significant difference between ML algorithm families (PTB: p = 0.33; stillbirth: p = 0.06) (Appendix pp 21–22). For SGA, the difference was borderline significant (p = 0.04), although this result may not be robust because of the small number of studies included (Appendix pp 21–22).

There were seven studies on PTB that conducted both development and external validation and reported the AUC, among which three used independent external datasets, and four performed temporal validation using data from the same site collected at different time periods. The median average AUC on external validation was 0.76 (range 0.63–0.91), indicating a very similar performance to that observed in the development datasets (p = 0.76, Appendix p 23).

The Egger’s test indicated evidence of publication bias for average AUCs for PTB (p = 0.04). The funnel plot showed clustering of studies in the upper-left region, corresponding to studies with larger sample size tending to report more modest AUCs. In leave-one-out sensitivity analysis, this was mainly influenced by Lee 2024. After removal of this study, Egger’s test was no longer significant (p = 0.37), although heterogeneity remained substantial (Appendix p 24). For best AUCs, a similar pattern of asymmetry was observed, however, the Egger’s test did not show statistical significance (p = 0.11, Appendix p 25). For SGA and stillbirth, the Egger’s tests and funnel plots did not show evidence of publication bias for either the best AUC or the average AUC (Appendix p 25), however, the number of included studies was too small to draw reliable conclusions.

## Discussion

Our review indicates that the overall performance of ML models based on routinely collected clinical data to predict adverse perinatal outcomes was moderate. Our review identified basic demographic variables and essential obstetric features of a woman’s history as the most frequently reported predictors, probably reflecting data availability. No specific or universally strong predictors were identified across models. In terms of prediction to enable early intervention, the second trimester may be the earliest time point which enables relatively good model performance and also allows sufficient time for intervention.

While ML classifiers have similar performance to logistic regression in most studies, a general trend towards improved predictive accuracy was observed. This tendency may be attributable to the inherent ability of ML algorithms to capture nonlinear relationships. The median number of predictors used in the best-performing ML models was only 22.5, suggesting that current perinatal datasets may lack the high-dimensional complexity which is usually present when ML demonstrates clear advantages.^112^ The limited performance may reflect the inherent limitations of the data used rather than the algorithms. In some comparative studies, logistic regression models included a large number of variables without strict controls for multicollinearity, potentially leading to overfitting and narrowing their performance gap with ML.^57^ Although ML did not demonstrate superior discriminative performance, it still offers practical advantages in model development, including greater tolerance of correlated predictors and less need for manual, stepwise variable selection.

Consistent with previous systematic reviews, current studies were inadequately reported, and demonstrated relatively low quality and high risk of bias. Furthermore, potential publication bias exists, raising the concern that selective reporting may hinder an accurate assessment of the true performance of ML methods. Therefore, methodologically rigorous studies are still needed to develop and validate the best-performing models, and progressively improve model robustness for clinical application.

This study has several strengths. To our knowledge, this is the largest and most up-to-date systematic review of ML models for predicting adverse perinatal outcomes using routinely collected clinical data. This review also represents the first application of the updated PROBAST + AI tool in perinatal ML research. Furthermore, while a review published in 2020 provided an initial quantitative comparison of ML to logistic regression, it was limited by high study heterogeneity.^14^ We aimed to reduce heterogeneity across studies by focusing on ML methods based on tabular data and limited our analysis to a few highly correlated perinatal outcomes, namely PTB, SGA and stillbirth.

However, our review also has some limitations. Firstly, although restrictive inclusion criteria were applied to reduce heterogeneity, substantial heterogeneity was found in our meta-analyses. Therefore, these findings should remain exploratory and be interpreted cautiously. A second limitation lies in the limited number of studies available for SGA and stillbirth, which reduced the robustness and prevented detailed comparison of algorithmic performance or network analysis. Finally, we focused exclusively on the most stable ML model types that most closely aligned with current clinical applicability. Therefore, this review is also restricted to studies focusing on structured clinical data-based prediction models that apply ML classifiers. We did not evaluate models mainly based on laboratory-specific indicators, and unstructured modalities such as medical imaging or large language models. Future studies should examine whether model performance varies by data source, predictor composition, and model development strategy. Our conclusion that conventional ML did not consistently outperform traditional logistic regression is specific to this data context. This finding challenges the incremental value of applying complex algorithms to low-dimensional clinical records but does not negate the potential of ML in other scenarios.

High risk of bias may lead to varying degrees of overestimation or misestimation of model performance, reduced reproducibility, and misleading conclusions about clinical applicability. Future models should be developed using standardised and transparent methodology following the PROBAST + AI and TRIPOD + AI guidelines to clarify whether rigorous ML models offer advantages. Furthermore, current unsatisfactory performance and no evidence of strong predictors suggested that models reliant on structured routine data may have reached their limits. One promising shift in direction is towards the integration of additional data sources, such as obstetric ultrasound metrics. Finally, current studies often train their model based on single site data or hospital-level electronic health records, which may capture institution-specific data patterns and risk factors, leading to the possibility of overfitting. However, few studies undergo external validation, leaving generalisability uncertain. In conclusion, despite substantial resource investment in model development, ML models showed only moderate performance and high risk of bias, which currently limits their clinical applicability. Future models will need more rigorous development, external validation, better calibration, and meaningful net benefits before they can be considered for routine use in perinatal care. At present, ML models may be more useful for risk stratification and hypothesis generation than for direct clinical implementation.

## Contributors

JZ developed the systematic review protocol, designed and conducted the scientific literature search, screened search results and assessed study eligibility, extracted data, assessed risk of bias, performed the analyses, interpreted the data, and drafted the first version of the manuscript.

ND, AT, XL, and AR screened search results, assessed study eligibility, extracted data, and assessed risk of bias.

SR contributed to data interpretation and manuscript revision.

MK conceived the study, contributed to the coordination of the study, assisted with the review protocol and literature search strategy, provided input on eligibility assessment, risk of bias assessment, data interpretation and manuscript revision.

JH conceived the study, contributed to the coordination of the study, assisted with the review protocol and literature search strategy, provided input on eligibility assessment, risk of bias assessment, data interpretation and manuscript revision.

JZ, MK and JH had full access to all data in the study, verified the data, and had final responsibility for the decision to submit the manuscript for publication. All authors reviewed and approved the final manuscript.

## Data sharing statement

All data were obtained from published studies, and will be made available upon request to the corresponding author.

## Declaration of interests

SR reports institutional grants from the Medical Research Council, Oxford University Hospital NHS Trust, Roche (R94776/CN002), and John Fell Fund, consulting fees from Lucem Health, and honoraria from the journal BMJ Heart. SR serves as Methodological Editor for BMJ Heart. MK reports institutional grants from National Institute for Health and Care Research, Healthcare Quality Improvement Partnership, and UKRI.
