# Supplementary material for: Machine learning to predict adverse perinatal outcomes: a systematic review and meta-analysis
Source: eClinicalMedicine. 2026 Aug 7;98:104138. doi: 10.1016/j.eclinm.2026.104138 (PMC13476551; doi:10.1016/j.eclinm.2026.104138)
Supplement: Supplementary file 1 [file mmc1.docx]

**Machine learning to predict adverse perinatal outcomes: a systematic review and analysis**

**Appendices**

**Table of Contents**

[Appendix 1. Pubmed search strategy for (preterm birth OR small for gestational age OR stillbirth) AND (machine learning OR prediction model)	2](#_Toc233108423)

[Appendix 2. EMBASE search strategy for (preterm birth OR small for gestational age OR stillbirth) AND (machine learning OR prediction model)	2](#_Toc233108424)

[Appendix 3. CINAHL search strategy for (preterm birth OR small for gestational age OR stillbirth) AND (machine learning OR prediction model)	3](#_Toc233108425)

[Appendix 4. Global Health search strategy for (preterm birth OR small for gestational age OR stillbirth) AND (machine learning OR prediction model)	4](#_Toc233108426)

[Appendix 5. Web of Science search strategy for (preterm birth OR small for gestational age OR stillbirth) AND (machine learning OR prediction model)	4](#_Toc233108427)

[Appendix 6. IEEE Xplore search strategy for (preterm birth OR small for gestational age OR stillbirth) AND (machine learning OR prediction model)	5](#_Toc233108428)

[Appendix 7. Additional characteristics of studies included in systematic review	7](#_Toc233108429)

[Appendix 8. PROBAST+AI risk of bias assessment of all included studies in each domain	10](#_Toc233108430)

[Appendix 9. Percentage of studies using each missing data handling method	13](#_Toc233108431)

[Appendix 10. Percentage of studies using each imbalanced data handling method	13](#_Toc233108432)

[Appendix 11. Percentage of studies using each data engineering method	14](#_Toc233108433)

[Appendix 12. Percentage of studies using each hyperparameter tuning method	14](#_Toc233108434)

[Appendix 13. Percentage of studies using each machine learning method	15](#_Toc233108435)

[Appendix 14. The number of predictors used in the best-performing machine learning models	15](#_Toc233108436)

[Appendix 15. Percentage of studies using each predictor type	16](#_Toc233108437)

[Appendix 16. Proportion of each predictor used in the best-performing models across 58 studies that reported predictor details and model AUCs	17](#_Toc233108438)

[Appendix 17. Average performance of machine learning models in each article based on internal validation	18](#_Toc233108439)

[Appendix 18. Comparison of prediction timing of all models and best-performing models across studies	18](#_Toc233108440)

[Appendix 19. Meta-analysis comparing machine learning and logistic regression for the prediction	19](#_Toc233108441)

[Appendix 20. Comparison of discriminative performance across machine learning algorithms	21](#_Toc233108442)

[Appendix 21. Average AUC of machine learning models for PTB based on external validation	23](#_Toc233108443)

[Appendix 22. Leave-one-out sensitivity analysis for Egger’s test and heterogeneity in average AUC of PTB	24](#_Toc233108444)

[Appendix 23. Funnel plot based on average and best AUCs	25](#_Toc233108445)

# **Appendix 1. Pubmed search strategy for (preterm birth OR small for gestational age OR stillbirth) AND (machine learning OR prediction model)**

1. (premature birth[MeSH Terms]) OR (obstetric labor, premature[MeSH Terms]) OR (infant, premature[MeSH Terms]) OR (infant, extremely premature[MeSH Terms])
2. (preterm[Title/Abstract] OR "pre-term"[Title/Abstract] OR premature[Title/Abstract]) AND (birth*[Title/Abstract] OR labour*[Title/Abstract] OR labor*[Title/Abstract] OR deliver*[Title/Abstract] OR infant*[Title/Abstract] OR baby[Title/Abstract] OR babies[Title/Abstract] OR neonate*[Title/Abstract])
3. prematurity[Title/Abstract] OR "gestational age at birth"[Title/Abstract] OR "gestational age at delivery"[Title/Abstract] OR preterms[Title/Abstract] OR "pre-terms"[Title/Abstract]
4. (infant, small for gestational age[MeSH Terms])
5. "small for gestational age"[Title/Abstract] OR "small for gestation"[Title/Abstract] OR "small for dates"[Title/Abstract] OR "small-for-dates"[Title/Abstract] OR "weight for dates"[Title/Abstract] OR "weight for gestational age"[Title/Abstract] OR "birthweight for dates"[Title/Abstract] OR "birthweight for gestational age"[Title/Abstract]
6. stillbirth[MeSH Terms]
7. stillbirth*[Title/Abstract] OR "still birth*"[Title/Abstract] OR stillborn*[Title/Abstract] OR "still born*"[Title/Abstract] OR abortion*[Title/Abstract] OR miscarriage*[Title/Abstract]
8. #1 OR #2 OR #3 OR #4 OR #5 OR #6 OR #7
9. (machine learning[MeSH Terms])
10. (machine[Title/Abstract] OR transfer[Title/Abstract] OR supervised[Title/Abstract] OR "semi supervised"[Title/Abstract] OR "semi-supervised"[Title/Abstract] OR active[Title/Abstract] OR unsupervised[Title/Abstract] OR self-supervised[Title/Abstract]) AND (learning[Title/Abstract] OR intelligence[Title/Abstract] OR algorithm*[Title/Abstract])
11. "data mining"[Title/Abstract] OR ((feature[Title/Abstract] OR pattern[Title/Abstract]) AND (detect*[Title/Abstract] OR extract*[Title/Abstract] OR ranking*[Title/Abstract] OR learning[Title/Abstract] OR recogni*[Title/Abstract]))
12. "hierarchical learning"[Title/Abstract]
13. (neural networks, computer[MeSH Terms])
14. "neural network*"[Title/Abstract] OR "connectionist model"[Title/Abstract] OR perceptron*[Title/Abstract]
15. support vector machine[MeSH Terms]
16. "support vector*"[Title/Abstract]
17. "random forest*"[Title/Abstract] OR "random decision forest*"[Title/Abstract] OR "decision tree*"[Title/Abstract]
18. boost*[Title/Abstract] OR CatBoost[Title/Abstract] OR AdaBoost[Title/Abstract] OR XGBoost[Title/Abstract] OR "bootstrap aggregat*"[Title/Abstract]
19. "stacked model"[Title/Abstract] OR stacking[Title/Abstract]
20. ((predict*[Title/Abstract] OR forecast*[Title/Abstract]) AND (model*[Title/Abstract] OR tool*[Title/Abstract] OR algorithm*[Title/Abstract])) OR "decision making"[Title/Abstract] OR decision-making[Title/Abstract]
21. #9 OR #10 OR #11 OR #12 OR #13 OR #14 OR #15 OR #16 OR #17 OR #18 OR #19 OR #20
22. #8 AND #21
23. ("2015/01/01"[Date - Publication] : "2026/01/17"[Date - Publication])
24. #22 AND #23

# **Appendix 2. EMBASE search strategy for (preterm birth OR small for gestational age OR stillbirth) AND (machine learning OR prediction model)**

1. exp prematurity/ or exp premature labor/
2. ((preterm or "pre-term" or premature) adj1 (birth$ or labour$ or labor$ or deliver$ or infant$ or baby or babies or neonate*)).ti,ab.
3. (prematurity or "gestational age at birth" or "gestational age at delivery" or preterms or "pre-terms").ti,ab.
4. exp small for gestational age /
5. ("small for gestational age" or "small for gestation" or "small-for-gestation" or "small for dates" or "small-for-dates" or "weight for dates" or "weight for gestational age" or "birthweight for dates" or "birthweight for gestational age").ti,ab.
6. exp stillbirth/
7. (stillbirth$ or stillborn$ or abortion$ or miscarriage$).ti,ab.
8. (still adj1 (born$ or birth$)).ti,ab.
9. 1 or 2 or 3 or 4 or 5 or 6 or 7 or 8
10. exp machine learning/
11. ((machine or transfer or supervised or "semi supervised" or "semi-supervised" or active or unsupervised or self-supervised) adj1 (learning or intelligence or algorithm$)).ti,ab.
12. ("data mining" or ((feature or pattern) adj1 (detect$ or extract$ or ranking$ or learning or recogni$))).ti,ab.
13. exp deep learning/
14. ("hierarchical learning").ti,ab.
15. exp artificial neural network/ or exp convolutional neural network/
16. ("neural network$" or "connectionist model" or perceptron$).ti,ab.
17. ("support vector$").ti,ab.
18. ("random forest$" or "random decision forest$" or "decision tree$").ti,ab.
19. (boost$ or CatBoost or AdaBoost or XGBoost or "bootstrap aggregate$").ti,ab.
20. ("stacked model" or stacking).ti,ab.
21. (((predict$ or forecast$) adj1 (model$ or tool$ or algorithm$)) or "decision making" or decision-making).ti,ab.
22. 12 or 13 or 14 or 15 or 16 or 17 or 18 or 19 or 20 or 21
23. 9 and 22
24. limit 23 to yr="2015 -2026"

# **Appendix 3. CINAHL search strategy for (preterm birth OR small for gestational age OR stillbirth) AND (machine learning OR prediction model)**

1. MH Childbirth, Premature+ OR MW Childbirth, Premature OR MH Infant, Premature+ OR MW Infant, Premature OR MH Labor, Premature+ OR MW Labor, Premature OR MH Outcomes of Prematurity+ OR MW Outcomes of Prematurity
2. (TI (preterm OR "pre-term" OR premature) N1 (labor* OR labour* OR birth OR deliver* OR infant) OR (AB (preterm OR "pre-term" OR premature) N1 (labor* OR labour* OR birth OR deliver* OR infant))
3. (TI prematurity OR "gestational age at birth" OR "gestational age at delivery" OR preterms OR"pre-terms") OR (AB prematurity OR "gestational age at birth" OR "gestational age at delivery" OR preterms OR"pre-terms")
4. MH Infant, Small for Gestational Age+ OR MW Infant, Small for Gestational Age
5. (TI "small for gestational age" OR "small for gestation" OR "small for dates" OR "small-for-dates" OR "weight for dates" OR "weight for gestational age" OR "birthweight for gestational age" OR "birth weight for dates") OR (AB "small for gestational age" OR "small for gestation" OR "small for dates" OR "small-for-dates" OR "weight for dates" OR "weight for gestational age" OR "birthweight for gestational age" OR "birth weight for dates")
6. (TI stillbirth* OR "still birth*" OR stillborn* OR "still born*" OR abortion* OR miscarriage*) OR (AB stillbirth* OR "still birth*" OR stillborn* OR "still born*" OR abortion* OR miscarriage*)
7. S1 OR S2 OR S3 OR S4 OR S5 OR S6
8. MH Machine Learning+ OR MW Machine Learning OR MH Data Mining+ OR MW Data Mining
9. (TI (machine OR transfer OR supervised OR "semi supervised" OR "semi-supervised" OR active OR unsupervised OR self-supervised) N1 (learning OR intelligence OR algorithm*)) OR (AB (machine OR transfer OR supervised OR "semi supervised" OR "semi-supervised" OR active OR unsupervised OR self-supervised) N1 (learning OR intelligence OR algorithm*))
10. (TI "data mining" OR ((feature OR pattern) N1 (detect* OR extract* OR ranking* OR learning OR recogni*))) or (AB "data mining" OR ((feature OR pattern) N1 (detect* OR extract* OR ranking* OR learning OR recogni*)))
11. (TI "hierarchical learning") OR (AB "hierarchical learning")
12. MW "Neural Networks (Computer)"+ AND MH "Neural Networks (Computer)"
13. (TI "neural network*" OR "connectionist model" OR perceptron*) OR (AB "neural network*" OR "connectionist model" OR perceptron*)
14. (TI "support vector*") OR (AB "support vector*")
15. (TI "random forest*" OR "random decision forest*" OR "decision tree*") OR (AB "random forest*" OR "random decision forest*" OR "decision tree*")
16. (TI boost* OR CatBoost OR AdaBoost OR XGBoost OR "bootstrap aggregate*") OR (AB boost* OR CatBoost OR AdaBoost OR XGBoost OR "bootstrap aggregate*")
17. (TI "stacked model" OR stacking) OR (AB "stacked model" OR stacking)
18. (TI ((predict* OR forecast*) N1 (model* OR tool* OR algorithm*)) OR "decision making" OR decision-making) OR (AB ((predict* OR forecast*) N1 (model* OR tool* OR algorithm*)) OR "decision making" OR decision-making)
19. S8 OR S9 OR S10 OR S11 OR S12 OR S13 OR S14 OR S15 OR S16 OR S17 OR S18
20. S7 AND S19
21. DT 20150101-20260117
22. S20 AND S21

# **Appendix 4. Global Health search strategy for (preterm birth OR small for gestational age OR stillbirth) AND (machine learning OR prediction model)**

1. exp prematurity/ or exp premature labor/
2. ((preterm or "pre-term" or premature) adj1 (birth$ or labour$ or labor$ or deliver$ or infant$ or baby or babies or neonate*)).ti,ab.
3. (prematurity or "gestational age at birth" or "gestational age at delivery" or preterms or "pre-terms").ti,ab.
4. exp small for gestational age /
5. ("small for gestational age" or "small for gestation" or "small-for-gestation" or "small for dates" or "small-for-dates" or "weight for dates" or "weight for gestational age" or "birthweight for dates" or "birthweight for gestational age").ti,ab.
6. exp stillbirth/
7. (stillbirth$ or stillborn$ or abortion$ or miscarriage$).ti,ab.
8. (still adj1 (born$ or birth$)).ti,ab.
9. 1 or 2 or 3 or 4 or 5 or 6 or 7 or 8
10. exp machine learning/
11. ((machine or transfer or supervised or "semi supervised" or "semi-supervised" or active or unsupervised or self-supervised) adj1 (learning or intelligence or algorithm$)).ti,ab.
12. ("data mining" or ((feature or pattern) adj1 (detect$ or extract$ or ranking$ or learning or recogni$))).ti,ab.
13. exp deep learning/
14. ("hierarchical learning").ti,ab.
15. exp artificial neural network/ or exp convolutional neural network/
16. ("neural network$" or "connectionist model" or perceptron$).ti,ab.
17. ("support vector$").ti,ab.
18. ("random forest$" or "random decision forest$" or "decision tree$").ti,ab.
19. (boost$ or CatBoost or AdaBoost or XGBoost or "bootstrap aggregate$").ti,ab.
20. ("stacked model" or stacking).ti,ab.
21. (((predict$ or forecast$) adj1 (model$ or tool$ or algorithm$)) or "decision making" or decision-making).ti,ab.
22. 12 or 13 or 14 or 15 or 16 or 17 or 18 or 19 or 20 or 21
23. 9 and 22
24. limit 23 to yr="2015 -2026"

# **Appendix 5. Web of Science search strategy for (preterm birth OR small for gestational age OR stillbirth) AND (machine learning OR prediction model)**

1. (TI=((premature OR "pre-term" OR preterm) AND (birth* OR labour* OR labor* OR infant OR deliver* OR infant* OR baby OR babies OR neonate*))) OR (AB=((premature OR "pre-term" OR preterm) AND (birth* OR labour* OR labor* OR infant OR deliver* OR infant* OR baby OR babies OR neonate*)))
2. (TI=(prematurity OR "gestational age at birth" OR "gestational age at delivery" OR preterms OR "pre-terms")) OR (AB=(prematurity OR "gestational age at birth" OR "gestational age at delivery" OR preterms OR "pre-terms"))
3. (TI=("small for gestational age" OR "small for gestation" OR "small for dates" OR "small-for-dates" OR "weight for dates" OR "weight for gestational age" OR "birthweight for dates" OR "birthweight for gestational age")) OR (AB=("small for gestational age" OR "small for gestation" OR "small for dates" OR "small-for-dates" OR "weight for dates" OR "weight for gestational age" OR "birthweight for dates" OR "birthweight for gestational age"))
4. (TI=(stillbirth* OR "still birth*" OR stillborn* OR "still born*" OR abortion* OR miscarriage*)) OR (AB=(stillbirth* OR "still birth*" OR stillborn* OR "still born*" OR abortion* OR miscarriage*))
5. #1 OR #2 OR #3 OR #4
6. (TI=((machine OR transfer OR supervised OR "semi supervised" OR "semi-supervised" OR active OR unsupervised OR self-supervised) AND (learning OR intelligence OR algorithm*))) OR (TI=((machine OR transfer OR supervised OR "semi supervised" OR "semi-supervised" OR active OR unsupervised OR self-supervised) AND (learning OR intelligence OR algorithm*)))
7. (TI=("data mining" OR ((feature OR pattern) AND (detect* OR extract* OR ranking* OR learning OR recogni*)))) OR (AB=("data mining" OR ((feature OR pattern) AND (detect* OR extract* OR ranking* OR learning OR recogni*))))
8. (TI=("deep learning" OR "hierarchical learning")) OR (AB=("deep learning" OR "hierarchical learning"))
9. (TI=(neural network* OR "connectionist model" OR perceptron*)) OR (AB=(neural network* OR "connectionist model" OR perceptron*))
10. (TI=("support vector*")) OR (AB=("support vector*"))
11. (TI=("random forest*" OR "random decision forest*" OR "decision tree*")) OR (AB=("random forest*" OR "random decision forest*" OR "decision tree*"))
12. (TI=(boost* OR CatBoost OR AdaBoost OR XGBoost OR "bootstrap aggregating")) OR (AB=(boost* OR CatBoost OR AdaBoost OR XGBoost OR "bootstrap aggregating"))
13. (TI=("stacked model" OR stacking)) OR (AB=("stacked model" OR stacking))
14. (TI=(((predict* OR forecast*) AND (model* OR tool* OR algorithm*)) OR "decision making" OR decision-making)) OR (AB=(((predict* OR forecast*) AND (model* OR tool* OR algorithm*)) OR "decision making" OR decision-making))
15. #6 OR #7 OR #8 OR #9 OR #10 OR #11 OR #12 OR #13 OR #14
16. #5 AND #15
17. DOP=(2015-01-01/2026-01-17)
18. #16 AND #17

# **Appendix 6.** **IEEE Xplore search strategy for (preterm birth OR small for gestational age OR stillbirth) AND (machine learning OR prediction model)**

1. ("Mesh_Terms":premature birth) OR ("Mesh_Terms":obstetric labor, premature) OR ("Mesh_Terms":infant, premature) OR ("Mesh_Terms":infant, extremely premature); Filters Applied: 01/01/2015 - 17/01/2026
2. (("Document Title":preterm OR "Document Title":"pre-term" OR "Document Title":premature) AND ("Document Title":birth* OR "Document Title":labour* OR "Document Title":labor* OR "Document Title":deliver* OR "Document Title":infant OR "Document Title":baby OR "Document Title":babies OR "Document Title":neonate*)) OR (("Abstract":preterm OR "Abstract":"pre-term" OR "Abstract":premature) AND ("Abstract":birth* OR "Abstract":labour* OR "Abstract":labor* OR "Abstract":deliver* OR "Abstract":infant OR "Abstract":baby OR "Abstract":babies OR "Abstract":neonate*)); Filters Applied: 01/01/2015 - 17/01/2026
3. ("Document Title":prematurity OR "Document Title":"gestational age at birth" OR "Document Title":"gestational age at delivery" OR "Document Title":preterms OR "Document Title":"pre-terms") OR ("Abstract":prematurity OR "Abstract":"gestational age at birth" OR "Abstract":"gestational age at delivery" OR "Abstract":preterms OR "Abstract":"pre-terms"); Filters Applied: 01/01/2015 - 17/01/2026
4. ("Mesh_Terms":Infant, Small for Gestational Age); Filters Applied: 01/01/2015 - 17/01/2026
5. ("Document Title":"small for gestational age" OR "Document Title":"small for gestation" OR "Document Title":"small for dates" OR "Document Title":"small-for-dates" OR "Document Title":"weight for dates" OR "Document Title":"weight for gestational age OR "Document Title":"birthweight for dates" OR "Document Title":"birthweight for gestational age") OR ("Abstract":"small for gestational age" OR "Abstract":"small for gestation" OR "Abstract":"small for dates" OR "Abstract":"small-for-dates" OR "Abstract":"weight for dates" OR "Abstract":"weight for gestational age OR "Abstract":"birthweight for dates" OR "Abstract":"birthweight for gestational age"); Filters Applied: 01/01/2015 - 17/01/2026
6. ("Mesh_Terms":Stillbirth); Filters Applied: 01/01/2015 - 17/01/2026
7. "Document Title":stillbirth* OR "Document Title":"still birth*" OR "Document Title":stillborn* OR "Document Title":"still born*" OR "Document Title":abortion* OR "Document Title":miscarriage*; Filters Applied: 01/01/2015 - 17/01/2026
8. "Abstract":stillbirth* OR "Abstract":"still birth*" OR "Abstract":stillborn* OR "Abstract":"still born*" OR "Abstract":abortion* OR "Abstract":miscarriage*; Filters Applied: 01/01/2015 - 17/01/2026

# **Appendix 7. Additional characteristics of studies included in systematic review**

| Author year | Study centre | Plurality | Parity | Interpretability | Clinical efficacy |
| --- | --- | --- | --- | --- | --- |
| Preterm birth | | | | | |
| Abdi et al (2025)^22^ | Single centre | Singleton | Analysed together | NA | NA |
| Abraham et al (2022)^23^ | Multi-centre | Singleton | Analysed separately | SHAP value | Decision curve analysis |
| Alif et al (2024)^24^ | NA | NA | NA | NA | NA |
| Alsaad et al (2022)^25^ | Multi-centre | NA | Analysed together | Code-level method | Decision curve analysis |
| Amini et al (2017)^26^ | Multi-centre | Analysed together | Analysed together | NA | Decision curve analysis |
| Begum et al (2021)^27^ | Multi-centre | NA | NA | NA | NA |
| Belaghi et al (2021)^28^ | Multi-centre | Singleton | Nulliparous | NA | NA |
| Belaghi et al (2024)^29^ | Multi-centre | Singleton | Multiparous | NA | NA |
| Bitar et al (2024)^30^ | Single centre | Analysed together | Analysed together | SHAP value | NA |
| Chen et al (2024)^31^ | Single centre | Singleton | Nulliparous | SHAP value | NA |
| Chun et al (2025)^32^ | Multi-centre | Analysed together | Analysed together | SHAP value | NA |
| Deshpande et al (2023)^33^ | Single centre | Singleton | Analysed together | NA | NA |
| Diaz et al (2022)^34^ | Single centre | Analysed together | Analysed together | NA | NA |
| Ding et al (2024)^35^ | Multi-centre | Analysed together | Analysed together | SHAP value | NA |
| Diniz et al (2025)^36^ | Multi-centre | Analysed together | Analysed together | Code-level method | NA |
| Ebrahimvandi et al (2022)^37^ | Multi-centre | Singleton | Analysed together | Partial dependence plots | NA |
| Esty et al (2018)^38^ | Multi-centre | Analysed together | Analysed separately | NA | NA |
| Farrokhi et al (2024)^39^ | Multi-centre | Analysed together | Analysed together | LIME | NA |
| Gao et al (2025)^40^ | Single centre | Singleton | Analysed together | SHAP value | NA |
| Hassan et al (2025)^41^ | Multi-centre | Analysed together | Analysed together | SHAP value | NA |
| Hershey et al (2022)^42^ | Multi-centre | NA | Analysed together | NA | NA |
| Huang et al (2024)^43^ | Multi-centre | Singleton | Nulliparous | NA | NA |
| Khan et al (2023)-a^44^ | Multi-centre | NA | Analysed separately | Code-level method | NA |
| Khan et al (2023)-b^45^ | Single centre | Singleton | Analysed separately | SHAP value, LIME | NA |
| Khatibi et al (2019)^46^ | Multi-centre | NA | NA | NA | NA |
| Kim et al (2024)^47^ | Multi-centre | Singleton | Nulliparous | Code-level method, Partial dependence plots, individual conditional expectation curve | NA |
| Kim et al (2025)-a^48^ | Multi-centre | Singleton | Nulliparous | SHAP value | NA |
| Kim (2025)-b^49^ | Multi-centre | Singleton | Nulliparous | NA | NA |
| Kloska et al (2025)^50^ | Single centre | Singleton | Analysed together | NA | NA |
| Koivu et al (2020)-a^51^ | Multi-centre | Singleton | Analysed together | NA | NA |
| Kong et al (2024)^52^ | Single centre | NA | NA | SHAP value | NA |
| Kokkinidis et al (2023)^53^ | Multi-centre | NA | NA | NA | NA |
| Lee et al (2019)^54^ | Single centre | Analysed together | Analysed together | SHAP value | NA |
| Lee et al (2020)^55^ | Single centre | Analysed together | Analysed together | NA | NA |
| Lee et al (2021)-a^56^ | Multi-centre | Singleton | Nulliparous | NA | NA |
| Lee et al (2021)-b^57^ | Multi-centre | Singleton | Nulliparous | NA | NA |
| Author year | **Study centre** | **Plurality** | **Parity** | **Interpretability** | **Clinical efficacy** |
| Lee et al (2022)-a^58^ | Multi-centre | Singleton | Nulliparous | NA | NA |
| Lee et al (2022)-b^59^ | Multi-centre | Singleton | Nulliparous | NA | NA |
| Lee et al (2024)^60^ | Multi-centre | Singleton | Nulliparous | NA | NA |
| Liu et al (2024)^61^ | Multi-centre | Singleton | Analysed together | Nomogram | Decision curve analysis |
| Marvin et al (2022)^62^ | NA | NA | NA | SHAP value | NA |
| Meem et al (2022)^63^ | Multi-centre | Analysed together | Analysed together | NA | NA |
| Mirzamoradi et al (2024)^64^ | Single centre | Analysed together | Analysed together | NA | NA |
| Padmavathi et al (2024)^65^ | NA | Analysed together | Analysed together | NA | NA |
| Pari et al (2017)^66^ | NA | NA | NA | NA | NA |
| Pari et al (2025)^67^ | Single centre | Analysed together | NA | NA | NA |
| Park et al (2024)^68^ | Single centre | Singleton | NA | SHAP value | NA |
| Pathak et al (2025)^69^ | NA | NA | NA | NA | NA |
| Pereira et al (2015)^70^ | Single centre | Analysed together | Analysed together | NA | NA |
| Pourahmad et al (2017)^71^ | Multi-centre | Analysed together | Analysed together | NA | NA |
| Priya et al (2025)^72^ | Single centre | Analysed together | Analysed together | NA | NA |
| Qian et al (2025)^73^ | Single centre | Analysed together | Analysed together | SHAP value | NA |
| Raja et al (2021)^74^ | Single centre | Singleton | Analysed together | NA | NA |
| Ramalingam et al (2019)^75^ | NA | NA | NA | NA | NA |
| Safi et al (2022)^76^ | Multi-centre | Analysed together | Analysed together | NA | NA |
| Siargkas et al (2025)^77^ | Single centre | Singleton | Analysed together | SHAP value | NA |
| Silva et al (2025)^78^ | Multi-centre | Analysed together | Analysed together | NA | NA |
| Song et al (2023)^79^ | Multi-centre | Singleton | Nulliparous | NA | NA |
| Sun et al (2022)^80^ | Single centre | Analysed together | Analysed together | NA | NA |
| Teng et al (2025)^81^ | Single centre | Singleton | Analysed together | SHAP value | Decision curve analysis |
| Tran et al (2016)^82^ | Single centre | Analysed together | Analysed together | NA | NA |
| Waynforth et al (2022)^83^ | Multi-centre | Analysed together | Analysed together | NA | NA |
| Wong et al (2022)^84^ | Multi-centre | Analysed together | Analysed separately | SHAP value | NA |
| Yang et al (2025)^85^ | Single centre | Singleton | Analysed together | SHAP value | Decision curve analysis |
| Yu et al (2024)^86^ | Multi-centre | Singleton | Analysed together | SHAP value | NA |
| Zhang et al (2022)^87^ | Single centre | Singleton | Analysed together | NA | NA |
| Zhang et al (2023)^88^ | Single centre | Analysed together | Analysed together | NA | NA |
| Zhang et al (2024)^89^ | Single centre | Analysed together | Analysed together | NA | NA |
| Small for gestational age | | | | | |
| Akhtar et al (2022)^90^ | Multi-centre | NA | NA | NA | NA |
| Brown et al (2025)^91^ | Multi-centre | Singleton | Nulliparous | NA | NA |
| Chen et al (2024)^92^ | Single centre | Singleton | Analysed together | NA | NA |
| Deng et al (2025)^93^ | Multi-centre | Singleton | Analysed together | NA | NA |
| Kuhle et al (2018)^94^ | Multi-centre | Singleton | Analysed separately | NA | NA |
| Saw et al (2021)^95^ | Single centre | Singleton | NA | NA | NA |
| Stoenescu et al (2021)^96^ | Single centre | Singleton | NA | NA | NA |
| Van et al (2024)-a^97^ | Single centre | Analysed together | NA | SHAP value, beeswarm plot | NA |
| Van et al (2024)-b^98^ | Single centre | Singleton | Analysed together | NA | NA |
| Yu et al (2025)^99^ | Multi-centre | Singleton | Analysed together | SHAP value | NA |
| Author year | **Study centre** | **Plurality** | **Parity** | **Interpretability** | **Clinical efficacy** |
| Zhang et al (2023)^100^ | Single centre | Singleton | NA | NA | NA |
| Adebanji et al (2024)^101^ | Single centre | Analysed together | Analysed together | NA | NA |
| Stillbirth |  |  |  |  |  |
| Aga et al (2025)^102^ | Single centre | Analysed together | Analysed together | SHAP value | NA |
| Bosschiete et al (2023)^103^ | Multi-centre | Singleton | Analysed together | Code-level method | NA |
| Ferro et al (2025)^104^ | Multi-centre | Analysed together | Analysed together | NA | NA |
| Gunenc et al (2025)^105^ | Single centre | Singleton | Analysed together | NA | NA |
| Khatibi et al (2021)^106^ | Multi-centre | Analysed together | Analysed together | NA | NA |
| Kim et al (2025)-c^107^ | Multi-centre | Singleton | Analysed separately | SHAP value | NA |
| Kintomonho et al (2025)^108^ | Multi-centre | Analysed together | Analysed together | NA | NA |
| Koivu et al (2020)-b^109^ | Multi-centre | Singleton | Analysed together | NA | NA |
| Koivu et al (2020)-a^51^ | Multi-centre | Singleton | Analysed together | NA | NA |
| Malacova et al (2020)^110^ | Multi-centre | Analysed together | Analysed together | NA | NA |
| Zhu et al (2026)^111^ | Single centre | Singleton | Analysed together | NA | NA |

Abbreviations: NA, not available.

# **Appendix 8. PROBAST+AI risk of bias assessment of all included studies in each domain**

|  | Model development | | | | | Model evaluation | | | | |
| --- | --- | --- | --- | --- | --- | --- | --- | --- | --- | --- |
| Author year | **Participants**  **& data sources** | **Predictors** | **Outcome** | **Analysis** | **Overall** | **Participants**  **& data sources** | **Predictors** | **Outcome** | **Analysis** | **Overall** |
| Preterm birth | | | | | | | | | | |
| Abdi et al (2025)^22^ | High | High | High | High | High | High | High | High | High | High |
| Abraham et al (2022)^23^ | High | Low | Low | High | High | High | Low | Low | High | High |
| Alif et al (2024)^24^ | High | High | High | High | High | High | High | High | High | High |
| Alsaad et al (2022)^25^ | High | Low | Low | High | High | High | Low | Low | High | High |
| Amini et al (2017)^26^ | Low | Low | Low | Unclear | Unclear | Low | Low | Low | High | High |
| Begum et al (2021)^27^ | High | Low | Low | High | High | High | Low | Low | High | High |
| Belaghi et al (2021)^28^ | High | Low | Low | Unclear | High | High | Low | Low | High | High |
| Belaghi et al (2024)^29^ | High | Low | Low | High | High | High | Low | Low | High | High |
| Bitar et al (2024)^30^ | Low | Low | Low | High | High | Low | Low | Low | High | High |
| Chen et al (2024)^31^ | High | Low | Low | High | High | High | Low | Low | High | High |
| Chun et al (2025)^32^ | High | Low | Low | High | High | High | Low | Low | High | High |
| Deshpande et al (2023)^33^ | High | Low | Low | High | High | High | Low | Low | High | High |
| Diaz et al (2022)^34^ | High | Low | Low | High | High | High | Low | Low | High | High |
| Ding et al (2024)^35^ | High | High | High | High | High | High | High | High | High | High |
| Diniz et al (2025)^36^ | High | High | High | Unclear | High | High | High | High | High | High |
| Ebrahimvandi et al (2022)^37^ | High | Low | Low | Unclear | High | High | Low | Low | High | High |
| Esty et al (2018)^38^ | High | Low | High | High | High | High | Low | High | High | High |
| Farrokhi et al (2024)^39^ | High | Low | Low | High | High | High | Low | Low | High | High |
| Gao et al (2025)^40^ | High | Low | Low | High | High | High | Low | Low | High | High |
| Hassan et al (2025)^41^ | High | Low | Low | High | High | High | Low | Low | High | High |
| Hershey et al (2022)^42^ | Low | Low | Low | High | High | Low | Low | Low | High | High |
| Huang et al (2024)^43^ | Low | Low | Low | Unclear | Unclear | Low | Low | Low | High | High |
| Khan et al (2023)-a^44^ | Low | High | High | High | High | Low | High | High | High | High |
| Khan et al (2023)-b^45^ | Low | High | High | High | High | Low | High | High | High | High |
| Khatibi et al (2019)^46^ | High | Low | Low | High | High | High | Low | Low | High | High |
| Kim et al (2024)^47^ | High | Low | High | High | High | High | Low | High | High | High |
| Kim et al (2025)-a^48^ | High | High | High | High | High | High | High | High | High | High |
| Kim (2025)-b^49^ | High | Low | Low | High | High | High | Low | Low | High | High |
| Kloska et al (2025)^50^ | High | High | High | High | High | High | High | High | High | High |
| Koivu et al (2020)-a^51^ | High | Low | Low | High | High | High | Low | Low | High | High |
| Kong et al (2024)^52^ | High | Low | High | High | High | High | Low | High | High | High |
| Kokkinidis et al (2023)^53^ | High | Low | Low | High | High | High | Low | Low | High | High |
| Lee et al (2019)^54^ | High | Low | High | High | High | High | Low | High | High | High |
| Lee et al (2020)^55^ | High | Low | High | Unclear | High | High | Low | High | High | High |
| Lee et al (2021)-a^56^ | High | Low | High | High | High | High | Low | High | High | High |
| Lee et al (2021)-b^57^ | High | High | High | High | High | High | High | High | High | High |
| Lee et al (2022)-a^58^ | High | Low | High | High | High | High | Low | High | High | High |
| Lee et al (2022)-b^59^ | High | Low | High | High | High | High | Low | High | High | High |
| Lee et al (2024)^60^ | High | Low | High | High | High | High | Low | High | High | High |
| Liu et al (2024)^61^ | High | High | High | High | High | High | High | High | High | High |
| Author year | **Participants**  **& data sources** | **Predictors** | **Outcome** | **Analysis** | **Overall** | **Participants**  **& data sources** | **Predictors** | **Outcome** | **Analysis** | **Overall** |
| Marvin et al (2022)^62^ | High | High | High | High | High | High | High | High | High | High |
| Meem et al (2022)^63^ | High | High | High | High | High | High | High | High | High | High |
| Mirzamoradi et al (2024)^64^ | High | Low | Low | High | High | High | Low | Low | High | High |
| Padmavathi et al (2024)^65^ | High | High | High | High | High | High | High | High | High | High |
| Pari et al (2017)^66^ | High | Low | High | High | High | High | Low | High | High | High |
| Pari et al (2025)^67^ | High | Low | Low | High | High | High | Low | Low | High | High |
| Park et al (2024)^68^ | Low | Low | High | High | High | Low | Low | High | High | High |
| Pathak et al (2025)^69^ | High | Low | Low | High | High | High | Low | Low | High | High |
| Pereira et al (2015)^70^ | High | Low | High | High | High | High | Low | High | High | High |
| Pourahmad et al (2017)^71^ | High | High | High | High | High | High | High | High | High | High |
| Priya et al (2025)^72^ | High | Low | Low | High | High | High | Low | Low | High | High |
| Qian et al (2025)^73^ | High | Low | High | High | High | High | Low | High | High | High |
| Raja et al (2021)^74^ | High | High | High | High | High | High | High | High | High | High |
| Ramalingam et al (2019)^75^ | High | Low | High | High | High | High | Low | High | High | High |
| Safi et al (2022)^76^ | High | Low | Low | High | High | High | Low | Low | High | High |
| Siargkas et al (2025)^77^ | High | Low | Low | High | High | High | Low | Low | High | High |
| Silva et al (2025)^78^ | High | Low | Low | High | High | High | Low | Low | High | High |
| Song et al (2023)^79^ | High | Low | High | High | High | High | Low | High | High | High |
| Sun et al (2022)^80^ | High | Low | Low | High | High | High | Low | Low | High | High |
| Teng et al (2025)^81^ | High | Low | Low | High | High | High | Low | Low | High | High |
| Tran et al (2016)^82^ | High | Low | Low | High | High | High | Low | Low | High | High |
| Waynforth et al (2022)^83^ | High | Low | Low | Unclear | High | High | Low | Low | High | High |
| Wong et al (2022)^84^ | High | High | High | High | High | High | High | High | High | High |
| Yang et al (2025)^85^ | High | Low | Low | High | High | High | Low | Low | High | High |
| Yu et al (2024)^86^ | High | High | High | High | High | High | High | High | High | High |
| Zhang et al (2022)^87^ | High | Low | High | High | High | High | Low | High | High | High |
| Zhang et al (2023)^88^ | High | Low | Low | High | High | High | Low | Low | High | High |
| Zhang et al (2024)^89^ | High | Low | Low | High | High | High | Low | Low | High | High |
| Small for gestational age | | | | | | | | | | |
| Akhtar et al (2022)^90^ | High | Low | Low | High | High | High | Low | Low | High | High |
| Brown et al (2025)^91^ | High | High | High | Unclear | High | High | High | High | Unclear | High |
| Chen et al (2024)^92^ | High | Low | Low | High | High | High | Low | Low | High | High |
| Deng et al (2025)^93^ | High | Low | Low | High | High | High | Low | Low | High | High |
| Kuhle et al (2018)^94^ | High | Low | Low | High | High | High | Low | Low | High | High |
| Saw et al (2021)^95^ | High | High | High | High | High | High | High | High | High | High |
| Stoenescu et al (2021)^96^ | Low | Low | Low | High | High | Low | Low | Low | High | High |
| Van et al (2024)-a^97^ | High | Low | Low | High | High | High | Low | Low | High | High |
| Van et al (2024)-b^98^ | High | High | High | High | High | High | High | High | High | High |
| Yu et al (2025)^99^ | High | Low | High | Low | High | High | Low | High | Low | High |
| Zhang et al (2023)^100^ | High | High | High | High | High | High | High | High | High | High |
| Stillbirth | | | | | | | | | | |
| Adebanji et al (2024)^101^ | High | High | High | High | High | High | High | High | High | High |
| Aga et al (2025)^102^ | High | High | High | High | High | High | High | High | High | High |
| Bosschiete et al (2023)^103^ | High | Low | Low | Low | High | High | Low | Low | High | High |
| Author year | **Participants**  **& data sources** | **Predictors** | **Outcome** | **Analysis** | **Overall** | **Participants**  **& data sources** | **Predictors** | **Outcome** | **Analysis** | **Overall** |
| Ferro et al (2025)^104^ | High | Low | Low | High | High | High | Low | Low | High | High |
| Gunenc et al (2025)^105^ | High | Low | Low | High | High | High | Low | Low | High | High |
| Khatibi et al (2021)^106^ | High | High | High | Unclear | High | High | High | High | High | High |
| Kim et al (2025)-c^107^ | High | Low | Low | Unclear | High | High | Low | Low | High | High |
| Kintomonho et al (2025)^108^ | High | High | High | High | High | High | High | High | High | High |
| Koivu et al (2020)-b^109^ | High | Low | Low | High | High | High | Low | Low | High | High |
| Koivu et al (2020)-a^51^ | High | Low | Low | High | High | High | Low | Low | High | High |
| Malacova et al (2020)^110^ | High | High | High | Unclear | High | High | High | High | High | High |
| Zhu et al (2026)^111^ | High | Low | Low | High | High | High | Low | Low | High | High |

# **Appendix 9. Percentage of studies using each missing data handling method**

Each study may use more than one method.

Abbreviations: PTB, preterm birth; SGA, small for gestational age.

# **Appendix 10. Percentage of studies using each imbalanced data handling method**

Each study may use more than one method.

Abbreviations: PTB, preterm birth; SGA, small for gestational age; SMOTE, synthetic minority over-sampling technique.

# **Appendix 11. Percentage of studies using each data engineering method**

Each study may use more than one method.

Abbreviations: PTB, preterm birth; SGA, small for gestational age.

# **Appendix 12. Percentage of studies using each hyperparameter tuning method**

Abbreviations: PTB, preterm birth; SGA, small for gestational age.

# **Appendix 13. Percentage of studies using each machine learning method**

Each study may use more than one method.

Abbreviations: PTB, preterm birth; SGA, small for gestational age.

# **Appendix 14. The number of predictors used in the best-performing machine learning models**

One study on PTB using 36,919 predictors is not shown in the figure due to scale distortion.

Abbreviations: PTB, preterm birth; SGA, small for gestational age

# **Appendix 15. Percentage of studies using each predictor type**

Each study may use more than one type of predictor.

Abbreviations: PTB, preterm birth; SGA, small for gestational age.

# **Appendix 16. Proportion of each predictor used in the best-performing models across 58 studies that reported predictor details and model AUCs**

# **Appendix 17. Average performance of machine learning models in each article based on internal validation**

Abbreviations: AUC, average area under the receiver operating characteristic curve; PTB, preterm birth; SGA, small for gestational age.

# **Appendix 18. Comparison of prediction timing of all models and best-performing models across studies**

| Timing of prediction | All models | Best-performing model ^*^ |
| --- | --- | --- |
| Early antenatal phase | 5.6% | 0% |
| First trimester | 22.2% | 11.1% |
| Second trimester | 28.9% | 20.0% |
| Third trimester | 12.2% | 11.1% |
| Around delivery | 27.8% | 26.7% |
| After birth | 13.3% | 11.1% |
| Unclear | 23.3% | 20.0% |

^*^If multiple best-performing models were reported at different prediction time points, the model with the earliest prediction time was included in this table.

# **Appendix 19. Meta-analysis comparing machine learning and logistic regression for the prediction**

| Outcome | Number of studies (k) | Pooled ΔAUC (95% CI) | p-value | I² (%) | I² interpretation | τ²  (95% PI) | |
| --- | --- | --- | --- | --- | --- | --- | --- |
| PTB | **33** | **0.03**  **(0.01, 0.06)** | **0.02** | **99.4** | **High** | **0.01**  **(-0.12, 0.18)** |  |
| SGA | **6** | **0.01**  **(-0.004, 0.03)** | **0.14** | **53.8** | **Substantial** | **0.0002**  **(-0.03, 0.05)** | |
| Stillbirth | **7** | **0.04**  **(-0.03, 0.09)** | **0.08** | **93.3** | **High** | **0.004**  **(-0.13, 0.22)** | |

Studies reporting AUCs for both ML and logistic regression models were included in this analysis. Squares represent study-specific effect estimates (ΔAUC = AUC_ML − AUC_LR) and horizontal lines indicate 95% confidence intervals. Diamonds represent pooled effect estimates from random-effects meta-analyses. Study-specific AUCs, effect estimates (95% confidence intervals), and study weights are displayed. Heterogeneity was assessed using I² and τ² statistics.

Abbreviations: AUC, area under the receiver operating characteristic curve; CI, confidence interval; LR, logistic regression; ML, machine learning; PTB, preterm birth; SGA, SGA, small for gestational age; RE, random-effects.

# **Appendix 20. Comparison of discriminative performance across machine learning algorithms**

| Outcome | Algorithm | Number of studies | Pooled AUC  (95% CI) | τ² between | τ² within | Omnibus Wald p-value |
| --- | --- | --- | --- | --- | --- | --- |
| PTB | Random forest | 30 | 0.79  (0.74-0.84) | **0.34** | **0.36** | **0.33** |
|  | Neural network | 21 | 0.75  (0.68-0.81) |  |  |  |
|  | Boost | 18 | 0.80  (0.74-0.85) |  |  |  |
|  | Decision tree | 14 | 0.77  (0.69-0.83) |  |  |  |
|  | Support vector machine | 12 | 0.78  (0.70-0.84) |  |  |  |
|  | Naive Bayes | 8 | 0.67  (0.56-0.77) |  |  |  |
|  | Ensemble | 5 | 0.81  (0.69-0.89) |  |  |  |
|  | Other ML | 11 | 0.78  (0.70-0.85) |  |  |  |
| SGA | Random forest | 4 | 0.81  (0.76-0.85) | **<0.0001** | **0.09** | **0.04** |
|  | Neural network | 3 | 0.71  (0.63-0.77) |  |  |  |
|  | Boost | 3 | 0.74  (0.67-0.80) |  |  |  |
| Stillbirth | Random forest | 5 | 0.86  (0.73-0.93) | **1.25** | **0.05** | **0.06** |
|  | Neural network | 4 | 0.79  (0.62-0.90) |  |  |  |
|  | Boost | 5 | 0.84  (0.70-0.92) |  |  |  |
|  | Decision tree | 4 | 0.82  (0.67-0.92) |  |  |  |

If a study reported multiple models using the same algorithm, only the model with the highest AUC was retained. Squares represent pooled AUC estimates and horizontal lines indicate 95% confidence intervals. Study number, pooled AUCs (95% confidence intervals), and between-study and within-study heterogeneity estimates (τ²) are displayed. Differences between algorithm families were assessed using an omnibus Wald test.

Abbreviations: AUC, area under the receiver operating characteristic curve; ML, machine learning; PTB, preterm birth; SGA, SGA, small for gestational age.

# **Appendix 21. Average AUC of machine learning models for PTB based on external validation**

For external validation group, triangular markers represent models evaluated using independent external datasets, and diamond-shaped markers indicate temporal validation.

Abbreviations: AUC-E, average area under the receiver operating characteristic curve based on external validation; AUC-I, average area under the receiver operating characteristic curve based on internal validation; PTB, preterm birth.

# **Appendix 22. Leave-one-out sensitivity analysis for Egger’s test and heterogeneity in average AUC of PTB**

A leave-one-out sensitivity analysis was conducted for average AUCs in PTB studies because Egger’s test suggested funnel plot asymmetry. Lee 2024 was identified as the main contributor to this asymmetry. After removing this study, Egger’s test was no longer significant (p = 0.37), although heterogeneity remained substantial.

# **Appendix 23. Funnel plot based on average and best AUCs**

Abbreviations: AUC, average area under the receiver operating characteristic curve; PTB, preterm birth; SGA, small for gestational age.
